# Expression of stem cell factor/c-kit signaling pathway components in diabetic fibrovascular epiretinal membranes

**Published:** 2010-06-15

**Authors:** Ahmed M. Abu El-Asrar, Sofie Struyf, Ghislain Opdenakker, Jo Van Damme, Karel Geboes

**Affiliations:** 1 Department of Ophthalmology, College of Medicine, King Saud University, Riyadh, Saudi Arabia; 2 Rega Institute for Medical Research, Laboratory of Molecular Immunology, Leuven, Belgium; 3 Laboratory of Histochemistry and Cytochemistry, University of Leuven, Leuven, Belgium

## Abstract

**Purpose:**

Stem cell factor (SCF)/c-kit signaling promotes recruitment of endothelial progenitor cells and contributes to ischemia-induced new vessel formation. We investigated the expression of the components of this pathway, including c-kit, SCF, granulocyte colony-stimulating factor (G-CSF), endothelial nitric oxide synthase (eNOS), and the chemokine receptor CXCR4, in proliferative diabetic retinopathy (PDR) epiretinal membranes.

**Methods:**

Membranes from eight patients with active PDR and 12 patients with inactive PDR were studied by immunohistochemistry.

**Results:**

Blood vessels expressed c-kit, SCF, G-CSF, eNOS, and CXCR4 in 18, 15, 19, 20, and 20 out of 20 membranes, respectively. Significant correlations were detected between the number of blood vessels expressing CD34 and the number of blood vessels expressing SCF (r=0.463; p=0.04), G-CSF (r=0.87; p<0.001), eNOS (r=0.864; p<0.001), and CXCR4 (r=0.864; p<0.001). Stromal cells expressed c-kit, SCF, eNOS, and CXCR4 in 19, 15, 20, and 20 membranes, respectively. The numbers of blood vessels expressing CD34 (p=0.005), c-kit (p=0.03), G-CSF (p=0.007), eNOS (p=0.001), and CXCR4 (p=0.018) and stromal cells expressing c-kit (p=0.013), SCF (p<0.001), eNOS (p=0.048), and CXCR4 (p=0.003) were significantly higher in active membranes than in inactive membranes.

**Conclusions:**

SCF/c-kit signaling might contribute to neovascularization in PDR.

## Introduction

Ischemia-induced retinal neovascularization in association with the outgrowth of fibrovascular epiretinal membranes at the vitreoretinal interface is a hallmark feature of proliferative diabetic retinopathy (PDR) and often leads to severe visual loss due to vitreous hemorrhage and/or tractional retinal detachment. Increasing evidence suggests that circulating bone marrow-derived endothelial progenitor cells (EPCs) home to the ischemic region, differentiate into mature endothelial cells in situ, and can contribute to the process of neovascularization [1].

Stem cell factor (SCF), or kit ligand, is a peptide growth factor that exists as a membrane-bound protein but may be cleaved by proteases, such as matrix metalloproteinase-9 (MMP-9), to produce a soluble cytokine [2,3]. SCF is important for the survival and differentiation of hematopoietic stem cells. The receptor for SCF, the proto-oncogene c-kit, is a tyrosine kinase that is expressed by bone marrow-derived endothelial stem/progenitor cells [4,5]. SCF ligand binding leads to phosphorylation and activation of the c-kit receptor and its downstream signaling proteins, which have been implicated in cell proliferation, cell adhesion, and cell survival as well as chemotaxis [3,6]. Several studies have demonstrated that SCF/c-kit signaling promotes the survival, migration differentiation, and capillary tube formation of endothelial cells [6,7] and plays an important role in ischemia-induced neovascularization [2,4,6,8-10].

The cytokine granulocyte colony-stimulating factor (G-CSF) has been shown to promote the mobilization of bone marrow-derived c-kit^+^ EPCs and to enhance neovascularization of ischemic tissue [11]. In addition, endothelial nitric oxide synthase (eNOS) is crucial for the recruitment of EPCs in the circulation from the bone marrow and for firm c-kit^+^ cell adhesion to the vascular endothelium. eNOS is also required for neovascularization in ischemic tissue [12-15]. Researchers have also reported that c-kit^+^ cells express the chemokine stromal cell-derived factor-1 (SDF-1) receptor CXCR4 and that SDF-1 induces their migration [12,13].

Understanding the molecular mechanisms involved in the pathologic neovascularization observed in PDR is important for identifying novel targets for anti-angiogenic therapy. Given the role of the SCF/c-kit signaling pathway in ischemia-induced neovascularization, we hypothesized that SCF/c-kit signaling may play a role in PDR. The aim of the present study was, therefore, to examine the expression of SCF, c-kit, G-CSF, eNOS, and CXCR4 in epiretinal membranes from patients with PDR. The level of vascularization in epiretinal membranes was determined by immunodetection of the panendothelial cell marker CD34.

## Methods

### Epiretinal membrane specimens

Epiretinal fibrovascular membranes were obtained from 20 patients with PDR during pars plana vitrectomy for the repair of traction retinal detachment or combined traction/ rhegmatogenous retinal detachment. Fourteen (70%) patients were male and 6 (30%) were female. The age ranged from 25 to 64 years, with a mean of 47.7±13.7 years. Fifteen (75%) patients had insulin-dependent diabetes mellitus, and 5 (25%) had non-insulin-dependent diabetes mellitus. Duration of diabetes ranged from 12 to 34 years with a mean of 22.1±6.9 years. Level of glycosylated hemoglobin ranged from 7.3% to 13.2% with a mean of 9.0±1.6%. Using the operating microscope, the clinical ocular findings were graded at the time of vitrectomy for the presence or absence of visible new vessels on the retina or optic disc. Patients with active PDR were graded as such on the basis of visible new vessels on the retina or optic disc. Their absence indicated involuted (inactive) PDR. Active PDR was present in eight patients, and inactive PDR was present in 12 patients. Membranes were fixed in 10% formalin solution and embedded in paraffin. The study was conducted according to the tenets of the Declaration of Helsinki, and informed consent was obtained from all patients. The study was approved by the Research Center, College of Medicine, King Saud University, Riyadh, Saudi Arabia.

### Immunohistochemical staining

Endogenous peroxidase was abolished with 2% hydrogen peroxide in methanol for 20 min, and nonspecific background staining was blocked by incubating the sections for 5 min in normal swine serum. For c-kit detection, antigen retrieval was performed by boiling the sections in 10 mM EDTA buffer (pH 9.0; Sigma-Aldrich, Bornem, Belgium) for 30 min. For CD34, SCF, G-CSF, and eNOS detection, antigen retrieval was performed by boiling the sections in 10 mM citrate buffer (pH 6.0) for 30 min. Subsequently, the sections were incubated with the monoclonal and polyclonal antibodies listed in Table 1. The optimal working concentration and incubation time for the antibodies were determined earlier in pilot experiments. For SCF, a second step was introduced using 1/20 rabbit antigoat peroxidase plus 1/10 normal human serum. The sections were then incubated for 30 min with immunoglobulin conjugated to peroxidase-labeled dextran polymer (EnVision [Flex]; Dako, Carpinteria, CA). The reaction product was visualized by incubation for 10 min in 0.05 M acetate buffer at pH 4.9 containing 0.05% 3-amino-9-ethylcarbazole (Sigma-Aldrich) and 0.01% hydrogen peroxide, resulting in bright-red immunoreactive sites or containing 3, 3′-diaminobenzidine (Dako) and hydrogen peroxide, resulting in brown immunoreactive sites. The slides were then faintly counterstained with Harris hematoxylin.

**Table 1:** Monoclonal and polyclonal antibodies used in this study.

**Primary antibody**	**Dilution**	**Incubation time**	Source*
Anti-CD34 (Clone My 10) (mc)	1/50	60 min	BD Biosciences
Anti-c- kit (Code A4502 (pc)	1/20	60 min	Dako
Anti-SCF (Catalogue No. AB-255-NA) (mc)	1/10	60 min	R&D Systems
Anti-G-CSF (FL-207) sc-13102 (pc)	1/200	60 min	Santa Cruz Biotechnology, Inc.
Anti-eNOS (Clone 3/eNOS/NOS Type III) (mc)	1/20	60 min	BD Biosciences
Anti-CXCR4 (Clone 44716) (mc)	1/100	60 min	R&D Systems

*Location of manufacturers: BD Biosciences, San Jose, CA; Dako, Glostrup, Denmark; R&D Systems, Abingdon, UK; Santa Cruz Biotechnology, Inc., Santa Cruz, CA. Abbreviations: mc represents monoclonal ; pc represents polyclonal.

To identify the phenotype of cells expressing c-kit or CXCR4, sequential double immunohistochemistry was performed as previously described [16].

Omission or substitution of the primary antibody with an irrelevant antibody of the same species and staining with chromogen alone were used as negative controls. Sections from patients with colorectal carcinoma were used as positive controls. Archived paraffin-embedded tissue sections from the control patients were obtained from patients treated at the University Hospital, University of Leuven, Belgium, in full compliance with tenets of the Declaration of Helsinki.

### Quantitation

Immunoreactive blood vessels and cells were counted in five representative fields, using an eyepiece calibrated grid in combination with a 40× objective. These representative fields were selected based on the presence of immunoreactive blood vessels and stromal cells. With this magnification and calibration, the blood vessels and cells present in an area of 0.33×0.22 mm were counted. Data were expressed as mean values ±standard deviation and analyzed by the Mann–Whitney test. Pearson correlation coefficients were computed to investigate the linear relationship between the variables investigated. A p value less than 0.05 indicated statistical significance. The BMDP 2007 statistical package (BMDP Statistical Software, Inc., Los Angeles, CA) was used for the statistical analysis.

## Results

### Immunohistochemical analysis

**Figure 1:**
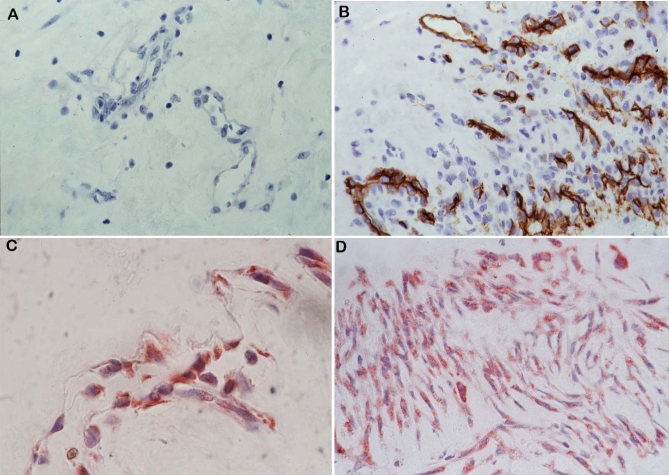
Negative control slide that was treated identically with an irrelevant antibody. No labeling was observed (**A**: original magnification 40×). Immunohistochemical staining for CD34. Blood vessels and stromal cells showed immunoreactivity for CD34 (**B**: original magnification 40×). Immunohistochemical staining for c-kit. Vascular endothelial cells (**C**: original magnification 100×) and stromal cells (**D**: original magnification 40×) showed immunoreactivity for c-kit.

**Figure 2:**
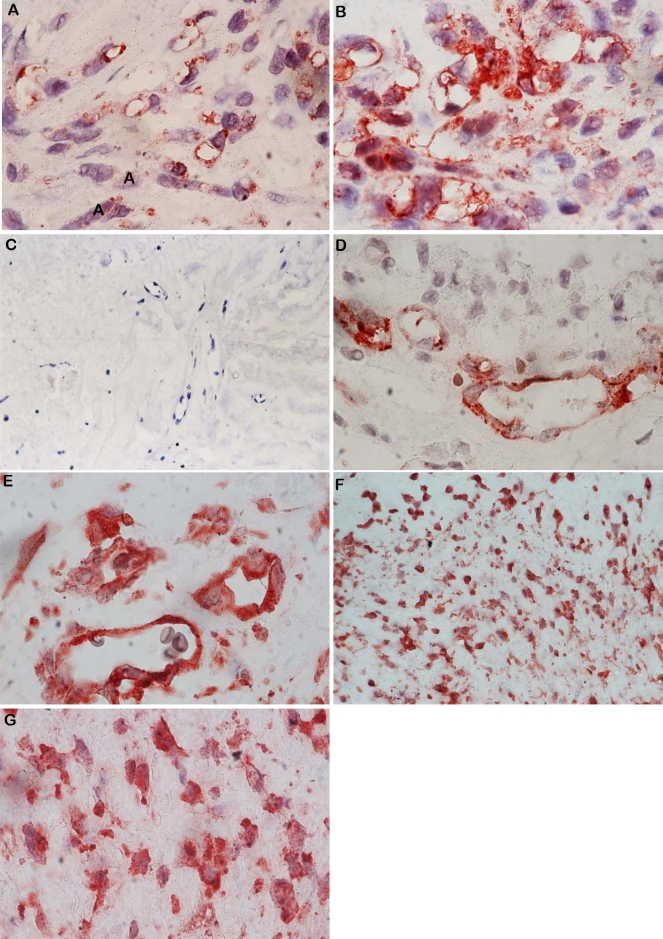
Immunohistochemical staining for stem cell factor (SCF). Vascular endothelial cells and stromal cells expressed strong immunoreactivity for SCF in a membrane from a patient with active proliferative diabetic retinopathy (PDR). **A**: Low power, original magnification 40×. **B**: high-power, original magnification 100×. SCF immunoreactivity is absent in a membrane from a patient with inactive PDR. Note that the membrane is composed mostly of fibrous tissue (**C**: original magnification 40×). Immunohistochemical staining for granulocyte colony-stimulating factor (G-CSF). G-CSF immunoreactivity was observed in vascular endothelial cells (**D**: original magnification 100×). Immunohistochemical staining for endothelial nitric oxide synthase (eNOS). Immunoreactivity for eNOS was observed in vascular endothelial cells (**E**: original magnification 100×) and stromal cells (**F**: Low-power, original magnification 40× and **G**: high-power, original magnification 100×).

**Figure 3:**
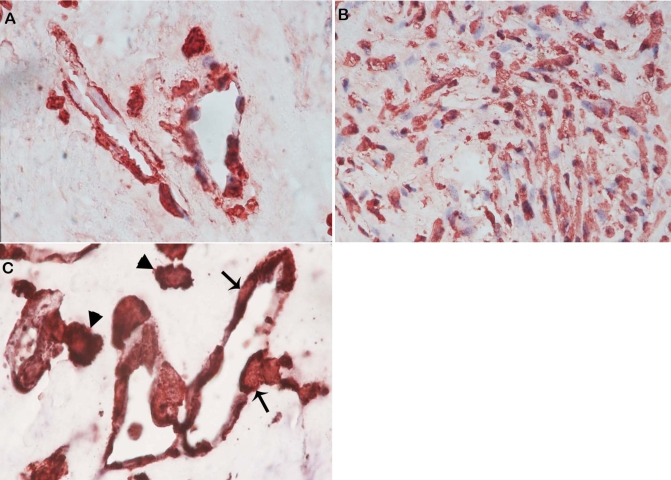
Immunohistochemical staining for CXCR4. Immunoreactivity for CXCR4 was observed in vascular endothelial cells (**A**: original magnification 100×) and stromal cells (**B**: original magnification 40×). Double immunohistochemistry for CXCR4 (red) and c-kit (blue). Cells co-expressing CXCR4 and c-kit were observed in the vascular endothelium (arrows) and in close association with blood vessels (arrowheads). **C**: original magnification 100×.

No staining was observed in the negative control slides (Figure 1A). All membranes showed blood vessels positive for the panendothelial cell marker CD34, with a mean number of 28.4±17.2 (range, 7–70). In addition, stromal cells expressing CD34 were noticed in close association with blood vessels (Figure 1B). Immunoreactivity for c-kit was present in 19 (95%) membranes. c-kit immunoreactivity was observed on vascular endothelial cells (Figure 1C) in 18 (90%) membranes, with a mean number of 9.1±6.7 (range, 0–25). Immunoreactivity for c-kit was also defined in stromal cells (Figure 1D) in 19 (95%) membranes, with a mean number of 24.6±32.1 (range, 0–140). Immunoreactivity for SCF was present in 15 (75%) membranes (Figure 2A-C). Strong SCF immunoreactivity was noticed on vascular endothelial cells (Figure 2A,B), with a mean number of 5.1±5.5 (range, 0–20). SCF immunoreactivity was also observed in stromal cells (Figure 2B), with a mean number of 11.6±9.3 (range, 0–30). Immunoreactivity for G-CSF was present in 19 (95%) membranes on vascular endothelial cells (Figure 2D), with a mean number of 10.4±8 (range, 0–28). There was no immunoreactivity for G-CSF in stromal cells. All membranes showed strong immunoreactivity for eNOS. Immunoreactivity for eNOS was visualized on vascular endothelial cells (Figure 2E), with a mean number of 22±10.3 (range, 4–40). Immunoreactivity for eNOS was also seen in stromal cells (Figure 2F,G), with a mean number of 28±21.8 (range, 5–100). Strong immunoreactivity for the chemokine receptor CXCR4 was present in all membranes. Immunoreactivity for CXCR4 was observed on vascular endothelial cells (Figure 3A) in 19 (95%) membranes, with a mean number of 21.2±12.6 (range, 0–55). Immunoreactivity for CXCR4 was also defined in stromal cells (Figure 3B) in all membranes, with a mean number of 69.8±46.4 (range, 12–150).

c-kit^+^, eNOS^+^, and CXCR4^+^ stromal cells were closely associated with the new vessels within the membranes. In serial sections, the distribution of cells expressing c-kit was similar to the distribution of cells expressing eNOS and CXCR4 (Figure 1D, Figure 2F, Figure 3B). Double immunohistochemistry confirmed that c-kit^+^ cells also co-expressed the chemokine SDF-1 receptor CXCR4 in the vascular endothelium and in the stroma (Figure 3C).

### Correlations and relationship with proliferative diabetic retinopathy activity

The mean number of blood vessels expressing CD34, c-kit, G-CSF, eNOS, and CXCR4 and stromal cells expressing c-kit, SCF, eNOS, and CXCR4 were significantly higher in membranes from patients with active PDR than in membranes from patients with inactive PDR (Table 2). Table 3 shows Pearson correlation coefficients between the numbers of the studied variables.

**Table 2:** Mean numbers of blood vessels and cells expressing various markers in relation to the type of proliferative diabetic retinopathy (PDR)

**Variable**	**Active PDR (n=8; Mean±S.D.)**	**Inactive PDR (n=12; Mean±S.D.)**	**p-value (Mann–Whitney test)**
Blood vessels expressing CD34	42.6±17.7	18.9±8.0	0.005*
Blood vessels expressing c-kit	13.1±6.9	6.4±5.3	0.03*
Cells expressing c-kit	42.1±43.8	12.9±13.6	0.013*
Blood vessels expressing SCF	7.8±7.1	3.3±3.3	0.128
Cells expressing SCF	20.4±5.0	5.8±6.2	<0.001*
Blood vessels expressing G-CSF	16.8± 8.6	6.1±3.8	0.007*
Blood vessels expressing eNOS	30.6±7.6	16.2±7.4	0.001*
Cells expressing eNOS	40.0±29.1	20.0±10.4	0.048*
Blood vessels expressing CXCR4	29.9±13.2	15.4±8.4	0.018*
Cells expressing CXCR4	107.5±36.5	44.7±33.8	0.003*

*Statistically significant at 5% level of significance.

**Table 3:** Pearson correlation coefficients

**Variable**	**Blood vessels expressing CD34**	**Cells expressing c-kit**	**Blood vessels expressing c-kit**	**Blood vessels expressing G-CSF**	**Cells expressing eNOS**	**Blood vessels expressing eNOS**	**Cells expressing SCF**	**Blood vessels expressing SCF**	**Cells expressing CXCR4**
**Cells expressing c-kit**									
r	0.11								
p	0.645								
**Blood vessels expressing c-kit**									
r	0.413	0.69							
p	0.07	0.001*							
**Blood vessels expressing G-CSF**									
r	0.871	-0.026	0.251						
p	<0.001*	0.912	0.286						
**Cells expressing eNOS**									
r	0.151	0.842	0.547	0.029					
p	0.525	<0.001*	0.013*	0.904					
**Blood vessels expressing eNOS**									
r	0.864	0.042	0.279	0.815	0.248				
p	<0.001*	0.861	0.234	<0.001*	0.293				
**Cells expressing SCF**									
r	0.591	0.527	0.573	0.436	0.552	0.632			
p	0.006*	0.017*	0.008*	0.055	0.012*	0.003*			
**Blood vessels expressing SCF**									
r	0.463	-0.047	0.109	0.28	-0.085	0.424	0.585		
p	0.040*	0.845	0.646	0.232	0.723	0.063	0.007*		
**Cells expressing CXCR4**									
r	0.431	0.373	0.179	0.355	0.521	0.625	0.722	0.596	
p	0.058	0.105	0.45	0.124	0.018*	0.003*	<0.001*	0.006*	
**Blood vessels expressing CXCR4**									
r	0.864	0.082	0.418	0.669	0.133	0.735	0.574	0.4	0.331
p	<0.001*	0.731	0.067	0.001*	0.577	<0.001*	0.008*	0.081	0.153

*Statistically significant at 5% level of significance. Where the row and column meet is the correlation coefficient and the p-value for the two variables.

## Discussion

In this immunohistochemical study of PDR membranes, the principal findings are that (a) PDR membranes showed immunoreactivity for SCF, c-kit, G-CSF, eNOS, and CXCR4 in vascular endothelial cells; (b) stromal cells expressed SCF, c-kit, eNOS, and CXCR4; (c) c-kit^+^ cells co-expressed the chemokine receptor CXCR4 and eNOS; (d) the number of blood vessels expressing CD34, c-kit, G-CSF, eNOS, and CXCR4 and the number of stromal cells expressing c-kit, SCF, eNOS, and CXCR4 in membranes from patients with active PDR were significantly higher than those in membranes from patients with inactive PDR; (e) there were significant correlations between the number of blood vessels expressing the panendothelial marker CD34 and the number of blood vessels expressing SCF, G-CSF, eNOS, and CXCR4 and the number of stromal cells expressing SCF. These data support the notion that bone marrow-derived cells contribute to neovascularization in PDR epiretinal membranes and that SCF/c-kit signaling may play a role in the pathogenesis of PDR.

Several studies have demonstrated that c-kit is expressed by bone marrow-derived progenitor cells that can give rise to endothelial cells [4,5]. Furthermore, bone marrow-derived c-kit-positive cells, but not c-kit-negative cells, have been reported to produce high levels of vascular endothelial growth factor (VEGF), differentiate into endothelial cells, and are incorporated into microvessels of the ischemic hindlimbs of mice [4]. Dentelli et al. [6] showed that activated microvascular endothelial cells challenged with interleukin-1β and tumor necrosis factor (TNF)-α express membrane-bound SCF and that recruitment of circulating EPCs depends on c-kit/membrane-bound SCF interaction. Moreover, in an in vivo model of angiogenesis, Dentelli et al. [6] demonstrated that c-kit /membrane-bound SCF interaction contributes to EPC recruitment to neovessels. Several studies have identified SCF/c-kit signaling as an important pathway involved in neovascularization. SCF/c-kit signaling promoted the survival, differentiation, migration, and capillary tube formation of endothelial cells [7,17] and induced new vessel formation in vivo [17]. Our findings confirm a previous study showing increased numbers of circulating mononuclear cells expressing c-kit in patients with diabetic retinopathy [18]. In addition, another study reported increased circulating EPCs expressing CD34/CD133 in patients with diabetic retinopathy [19]. Taken together, these findings suggest that circulating EPCs are involved in the progression of diabetic retinopathy.

Several studies have demonstrated that chronic, low-grade subclinical inflammation is implicated in the pathogenesis of diabetic retinopathy [20] and that leukocytes, in particular macrophages, are present in diabetic fibrovascular epiretinal membranes [21-23]. In a previous study, we demonstrated that the number of leukocytes in PDR membranes correlated significantly with the number of blood vessels expressing CD34, VEGF, and angiopoietin-2 and that the number of leukocytes in PDR membranes from patients with active PDR was significantly higher than that in membranes from patients with inactive PDR [21]. In addition, strong evidence indicates that macrophages play a critical role in the angiogenic process in the retina and choroid by secreting pro-inflammatory cytokines, such as interleukin-1β and TNF-α, that may affect endothelial cell functions, including proliferation, migration, and activation [24-26]. These findings suggest that bone marrow-derived cells could also indirectly contribute to neovascularization in PDR epiretinal membranes.

In the present study, we demonstrated the expression of SCF and c-kit by vascular endothelial cells and stromal cells in PDR membranes. The expression of both SCF and c-kit by vascular endothelial cells suggests an autocrine signaling in the propagation of neovascularization. Our observations are consistent with previous reports showing upregulation of SCF in ischemic tissues [2,10] by vascular endothelial cells [2]. The upregulation of SCF was coincident with infiltration of bone marrow-derived c-kit^+^ cells [2]. Bosch-Marce et al. [10] showed that the transcription factor hypoxia-inducible factor-1α is involved in ischemia-induced SCF upregulation. In a previous study, we demonstrated that vascular endothelial cells in PDR membranes expressed hypoxia-inducible factor-1α [21]. Our present study supported a previous study showing that ischemia-induced new vessels in the mesentery of diabetic rats expressed c-kit [9]. In addition, c-kit is expressed by newly formed vessels in ischemic limbs of patients treated with bone marrow-derived mononuclear cell therapy [8].

The cytokine G-CSF activates in vitro endothelial cell proliferation and shows angiogenic activity in vivo [27]. In addition, several studies have demonstrated that G-CSF recruits bone marrow-derived c-kit^+^ EPCs, which differentiate into endothelial cells, incorporate into the endothelium in vivo, and enhance neovascularization of ischemic tissue [11,28]. The neovascularization-promoting effect of G-CSF is also mediated by augmented VEGF secretion from neutrophils [11]. In the present study, vascular endothelial cells in PDR membranes expressed both SCF and G-CSF. Toth et al. [29] demonstrated that the combination of SCF/G-CSF has a synergistic effect on the promotion of chemotaxis of bone marrow-derived endothelial precursors and induction of bone marrow-derived neovascularization.

In the present study, c-kit^+^ cells in the vascular endothelium and in the stroma co-expressed the chemokine receptor CXCR4. CXCR4 is one of the two receptors for the chemokine SDF-1 [30]. Recently, several studies demonstrated that the majority of bone marrow and peripheral blood c-kit^+^ cells express the chemokine receptor CXCR4 and that SDF-1 induces their migration in vitro [12,13] and enhances venular rolling of c-kit^+^ cells in vivo [12]. In a previous study, we demonstrated that SDF-1 protein was specifically localized in vascular endothelial cells in PDR membranes [16]. Dutt et al. [31] demonstrated the cooperativity between SCF and SDF-1 in enhancing the chemotaxis of hematopoietic progenitor cells expressing both the CXCR4 and c-kit receptors. In addition, Tan et al. [32] showed that the combination of progenitor cell mobilization with G-CSF and enhanced homing of EPCs by SDF-1 promotes neovascularization in ischemic tissue. In the present study, the chemokine receptor CXCR4 was expressed by vascular endothelial cells in PDR membranes. Kaminski et al. [12] showed that stimulation with SDF-1 and TNF-α induces the expression of CXCR4 on the endothelium and that CXCR4 receptor expression participates in the firm adhesion of c-kit^+^ cells to the endothelium in vivo. They also described that eNOS is a crucial and specific factor for firm SDF-1/CXCR4-mediated c-kit^+^ cell adhesion to the vascular endothelium. In addition, other studies have demonstrated that eNOS is involved in SDF-1/CXCR4-mediated migration of EPCs [13] and that eNOS is also required for neovascularization in ischemic tissue [14,15]. In the present study, our analysis indicated that the number of stromal cells expressing the chemokine receptor CXCR4 was higher than the number of stromal cells expressing c-kit, SCF, and eNOS. The chemokine receptor CXCR4, in contrast to most chemokine receptors, is expressed on almost any cell type [33,34]. Consistent with these studies, we showed in a previous study the presence of cells co-expressing CXCR4 and the myofibroblast marker α-smooth muscle actin in proliferative vitreoretinopathy epiretinal membranes [35].

Several studies have demonstrated that MMP-9 is required for the mobilization and recruitment of bone marrow-derived c-kit^+^ endothelial progenitors to ischemic tissue and is essential for ischemia-induced neovascularization. MMP-9 activity is responsible for the cleavage of membrane SCF into soluble SCF, which is then available to bind its receptor c-kit. The resulting phosphorylation of the c-kit receptor corresponds to the mobilization of peripheral EPCs [2,3]. In a previous study, we demonstrated the presence of immunoreactivity for MMP-9 in myofibroblasts and in vascular endothelial cells in PDR membranes [36]. In addition, in situ zymography confirmed the presence of intense gelatinolytic activity in vascular endothelial cells and in scattered cells in PDR membranes [36]. Taken together, these findings suggest that MMP-9 is available and can promote the release of soluble SCF enhancing the recruitment of c-kit^+^ EPCs into PDR membranes.

In conclusion, our data suggest that bone marrow-derived c-kit^+^ cells contribute to new vessel formation in PDR fibrovascular membranes and that SCF/c-kit signaling might contribute to pathological neovascularization in PDR. This pathway may provide a promising molecular target for anti-angiogenic therapy.
